# Risk Factors and Symptoms of Meibomian Gland Loss in a Healthy Population

**DOI:** 10.1155/2016/7526120

**Published:** 2016-11-14

**Authors:** Anna Machalińska, Aleksandra Zakrzewska, Krzysztof Safranow, Barbara Wiszniewska, Bogusław Machaliński

**Affiliations:** ^1^Department of Histology and Embryology, Pomeranian Medical University, Al. Powstancow Wlkp. 72, 70-111 Szczecin, Poland; ^2^Department of Ophthalmology, Pomeranian Medical University, Al. Powstancow Wlkp. 72, 70-111 Szczecin, Poland; ^3^Department of Biochemistry and Medical Chemistry, Pomeranian Medical University, Al. Powstancow Wlkp. 72, 70-111 Szczecin, Poland; ^4^Department of General Pathology, Pomeranian Medical University, Al. Powstancow Wlkp. 72, 70-111 Szczecin, Poland

## Abstract

*Purpose*. The aim of this study was to investigate the relationships between MGL and ocular symptoms, several systemic conditions, and key markers of ocular surface health.* Methods*. We included into the study 91 healthy volunteers between the ages of 20 and 77 years. We analyzed meibomian gland morphology, function, and lid margin alterations. We correlated our findings with self-reported ocular symptoms, systemic medical history, lifestyle factors, and tear film abnormalities.* Results*. We observed that a high ocular surface disease index, a history of either chalazion or hordeolum, experience of puffy eyelids upon waking, and foreign body sensation all appeared to be predictors of an abnormal meiboscore after adjusting for age and sex (*p* = 0.0007; *p* = 0.001; *p* = 0.02; *p* = 0.001, resp.). Multivariate logistic regression model including age and sex showed that there were three independent predictors of abnormal meiboscore: older age (OR = 1.03, 95% CI = 1.01–1.04 per year, *p* = 0.006), postmenopausal hormone therapy (OR = 4.98, 95% CI = 1.52–16.30, *p* = 0.007), and the use of antiallergy drugs (OR = 5.85, 95% CI = 2.18–15.72, *p* = 0.0004).* Conclusion*. Our findings extend current knowledge on the pathophysiology of MGL.

## 1. Introduction

Meibomian gland dysfunction (MGD) is the most common cause of evaporative dry eye [1]. The meibomian glands represent large sebaceous glands placed in the tarsal plates of the eyelids and produce the lipids of the outermost layer of the preocular tear film [2]. The International Workshop on Meibomian Gland Dysfunction defined MGD as a chronic, diffuse abnormality of the meibomian glands commonly characterized by terminal duct obstruction and/or qualitative/quantitative changes in glandular secretion. These changes may result in an alteration of the tear film, symptoms of eye irritation, clinically apparent inflammation, and ocular surface disease [3]. Many ophthalmic and systemic factors, such as contact lens wear, hormonal disturbances, and skin diseases, as well as environmental and medicinal factors, contribute to the development of MGD [1, 4–7]. However, the pathogenesis of MGD is still poorly understood, and treatment options remain limited. It is widely accepted that hyperkeratinization and increased viscosity of the meibum represent the core pathogenic factors in the development of MGD. These factors lead to several downstream events, such as increased pressure within the ducts, resultant dilatation, and eventual acinar atrophy, the latter of which represents an advanced stage of MGD [2]. Atrophic degeneration of the meibomian glands is clinically less apparent and conceivably underreported unless more sophisticated methods such as meibography are applied.

The aim of this study was to characterize the prevalence of meibomian gland dropout in a healthy population and to explore the relationships between meibomian gland loss, ocular symptoms, and key markers of ocular surface health. We also aimed to analyze the influence of several systemic conditions on meibomian gland atrophy.

## 2. Methods

Ninety-one healthy volunteers (182 eyes) between the ages of 20 and 77 years were included in the study with an average age of 48.9 years. Participants were recruited from the staff of Pomeranian Medial University. Subjects were excluded from the study if they exhibited any active infection of the eye or active ocular allergy, had any evidence of lid deformity or abnormal lid movement disorder, or had undergone eye surgery within 1 year of the study visit. Moreover, exclusion criteria included skin diseases, contact lens wear, and continuous eye drop use (except artificial tears). Written informed consent was obtained from all subjects before examination. The study was approved by the Institutional Review Board of Pomeranian Medical University and adhered to the tenets of the Declaration of Helsinki [8].

A structured questionnaire was administered by a trained physician and included (1) self-reported ocular symptoms measured using the Ocular Surface Disease Index (OSDI) [9], (2) systemic medical history data (e.g., hypertension, diabetes mellitus, ischemic heart disease, thyroid disease, and current medications use), and (3) lifestyle factors (e.g., cigarette smoking, the frequency of using a computer, and predominantly indoor or outdoor occupational activity). Moreover all patients were questioned regarding the presence of the following ocular symptoms: dryness, foreign body sensation, pain, ocular fatigue, blurred vision, discharge, epiphora, puffy eyelids on waking, sticky sensation, and history of chalazion or hordeolum. Concurrently, when a respondent indicated the presence of one or more of the above symptoms, they were asked to specify when the symptoms were experienced: on waking, at evening, or during all day. Presence of each symptom was assigned to both eyes of a patient.

The examination included several steps as we described previously [10] and was performed sequentially as follows: measurement of the conversational blink rate, slit-lamp examination (including fluorescein staining of the ocular surface), tear film break-up time (TBUT) testing, the Schirmer test, quantification of morphologic lid features, examination of meibum expressibility/quality, and a meibography. TBUT was estimated by placing a single fluorescein strip over the inferior tear meniscus after instilling one drop of saline [11]. The Schirmer test was carried out without topical anesthesia. Lid margin abnormalities (LAS) were scored as 0 (absent) or 1 (present) for the following parameters: narrowed meibomian gland orifices, plugged meibomian gland orifices, posterior displacement of the orifices, lid margin telangiectasia, posterior lid margin hyperemia, rounding of the posterior margin, notching of the lid margin, eyelash loss, and trichiasis. Subsequently, the lid margin abnormality score was calculated according to the number of these abnormalities present in each eye.

The Meibum Quality Score (MQS) was graded as proposed by Tomlinson et al. [12]. Briefly, to assess obstruction of the MG orifices, digital pressure was applied to the lower tarsus, and the quality of meibum was scored semiquantitatively in central 8 glands as follows: grade 0, clear fluid; grade 1, cloudy fluid; grade 2, cloudy particulate fluid; and grade 3, inspissated, like toothpaste. Accordingly, the Meibum Expressibility Score (MES) was graded as follows: grade 0, all glands expressible; grade 1, 3-4 glands expressible; grade 2, 1-2 glands expressible; grade 3, no glands expressible.

**Figure 1:**
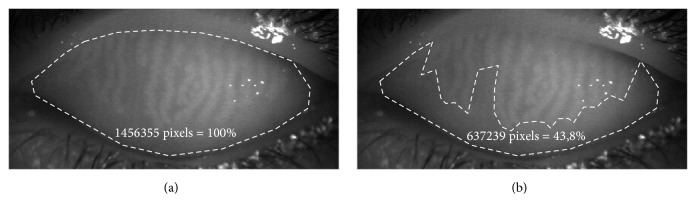
Definition of total area of the upper tarsus (a) and area of meibomian gland loss (b) on which subjective and computerized grading was based.

**Figure 2:**
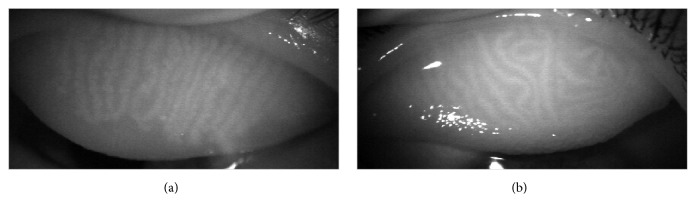
Representative cases of meibomian gland distortion. (a) No distortion. (b) Distortion: more than 50% of the meibomian glands changed in shape (distortion of >45°).

Meibography was performed using a BG-4M Noncontact Meibography System (Topcon Corp, Tokyo, Japan). All images were captured at 10x slit-lamp magnification. Meibomian gland loss [MGL] was calculated using ImageJ software and was defined as the proportion of the area of MGL in its relation to the total area of the upper tarsus (Figure 1). Subsequently, relative meiboscore was classified using a four-grade scale: 0, no MGL; 1, <33% of dropout area; 2, 33–66% of dropout area; and 3, >66% of dropout area. The presence of distortion was determined when distortion of >45° in meibomian gland was confirmed by meibography (Figure 2). Meibomian gland distortion was scored as 0 (absent) or 1 (present) as follows: 0 to indicate less than 50% of the meibomian glands had changed in shape (wrapped or twisted) and 1 to indicate more than 50% of the meibomian glands had changed in shape. Meibomian gland density was counted as the number of glands in one centimeter of the middle part of the upper eyelid.

Statistical analysis was performed with *n* = 182 eyes (each eye of a subject was treated separately). Because the distributions of most quantitative variables (including all meibomian gland outcome measures) were significantly different from normal distribution (as assessed by the Shapiro-Wilk's test), nonparametric tests were used. Mann-Whitney test was used for comparisons between groups and Spearman rank correlation coefficient (Rs) was calculated to measure strength of correlations between parameters. Multivariate logistic regression analysis adjusted for age and sex was performed to find independent predictors of abnormal meiboscore. *p* < 0.05 was considered statistically significant.

## 3. Results

### 3.1. Changes in Meibomian Glands and Association with Aging and Sex

The average age of participants in the sample was 48.9 ± 15 years. The study included 26 males and 65 females. The overall extent of MGL in our population ranged from 4.97 up to 70.7%. We noted a positive correlation between patient age and MGL (Rs = +0.28; *p* = 0.0001). This implies that percentage of MG dropout area increased gradually with age. Interestingly, we observed no differences in MGL between males and females (*p* = 0.97). Remarkably, the meibomian gland density did not correlate with age (Rs = +0.05; *p* = 0.52) or differ between males and females (*p* = 0.06). Additionally, we observed no differences in age or sex between eyes with distorted glands and those with no distortion (data not shown). Interestingly, we found higher MQS in females than in males (median: 1 versus 0, *p* < 0.001). Similarly, MES values were higher in females than in males (median: 1 versus 0, *p* = 0.002). This indicates that sex influences meibomian gland function.

### 3.2. Analysis of Ocular Symptoms and Their Correlations with Meibomian Gland Loss

Next, we focused on evaluating self-reported dry eye symptoms and tear film characteristics in study subjects. We observed that MGL positively correlated with the OSDI (Rs = +0.22, *p* = 0.003), and OSDI appeared to be an independent predictor of an abnormal meiboscore (stage 2 and higher) [11] after adjusting for age and sex (OR = 1.08 per OSDI point, 95% CI = 1.03–1.12, *p* = 0.0007). Accordingly, we observed a positive correlation between the OSDI and MES (Rs = +0.22; *p* = 0.002), as well as between OSDI and MQS (Rs = +0.24; *p* = 0.001), indicating that the OSDI questionnaire might be useful in diagnosing MGD.

Because the OSDI questionnaire does not differentiate evaporative dry eye disease from aqueous deficiency, we attempted to define eye symptoms related to MGD. To obtain more specific characteristics of clinical symptoms indicating the loss of meibomian gland tissue, we analyzed specific symptoms reported by the patient and their association with meibomian gland dropout. We observed that a history of chalazion or hordeolum, experience of puffy eyelids upon waking, and foreign body sensation appeared to be independent predictors of an abnormal meiboscore (stage 2 and higher) [12] after adjusting for age and sex (Table 1).

Interestingly, no correlation either between MGL and TBUT (Rs = −0.09; *p* = 0.21) or between MGL and Schirmer test values (Rs = −0.12; *p* = 0.10) was observed, suggesting that BUT and the Schirmer test are not key indicators for meibomian gland dropout.

### 3.3. Analysis of Risk Factors of Meibomian Gland Loss

Because there are available data suggesting that MGL may be associated with systemic factors, we assessed the impact of the abovementioned coincidence on meibomian gland tissue loss. Consequently, we evaluated the effect of underlying systemic disease, patient smoking status, and medications use on the extent of meibomian tissue dropout. The age- and sex-adjusted odds ratios (ORs) for the association of meiboscore with systemic factors are presented in Table 2.

We observed that participants on antiallergy drugs were more likely to have abnormal meiboscore (*p* = 0.0002). Accordingly, women treated with postmenopausal hormone therapy were found to have higher MGL compared with untreated women, and the use of hormone replacement therapy appeared to be an independent predictor of the abnormal meiboscore after adjusting for age and sex (*p* = 0,002). Similarly, smoking increased the likelihood of an abnormal meiboscore (OR = 2.05, 95% CI: 1.01–4.14; *p* = 0.04). Remarkably, no association was observed with systemic diseases such as hypertension, diabetes mellitus, heart disease, or thyroid disease. Subsequently, we assessed the effect of environmental factors on MGL. Controlling for age and sex the range of MG dropout appeared to be unaffected by either frequency of computer usage, predominantly indoor or outdoor occupational activity, or exposure to air conditioning (Table 2). Multivariate logistic regression model including age and sex showed that there were three independent predictors of abnormal meiboscore: older age (OR = 1.03, 95% CI = 1.01–1.04 per year, *p* = 0.006), postmenopausal hormone therapy (OR = 4.98, 95% CI = 1.52–16.30, *p* = 0.007), and the use of antiallergy drugs (OR = 5.85, 95% CI = 2.18–15.72, *p* = 0.0004).

### 3.4. Correlations between Functional and Morphological Meibomian Gland Parameters

Subsequent correlation analysis of the meibography images with the meibum quality/expressibility scores showed positive associations between the morphological and functional MG parameters. We observed a positive correlation between the MGL and MES (Rs = +0.20; *p* = 0.009), as well as between MGL and MQS (Rs = +0.20; *p* = 0.006). Moreover, an even stronger positive relationship was revealed between the MES and MQS (Rs = +0.50; *p* < 0.0001). This may indicate that qualitative and quantitative changes in the meibomian gland secretion resulting in its stagnation inside the glands lead to the loss of glandular tissue. Remarkably, we did not observe a correlation between MGL and LAS (Rs = +0.10; *p* = 0.20), suggesting that atrophy of meibomian gland tissue is not necessarily accompanied with the clinical signs of lid margin inflammation.

Next, we performed an extensive evaluation of the meibomian gland morphology parameters and analyzed the associations between meibomian gland loss, meibomian gland density, and the meibomian gland distortion scores. Interestingly, we observed no correlation between MGL and the meibomian gland density (Rs = −0.08; *p* = 0.28). Accordingly, the meibomian gland density was not correlated with ocular symptoms (Rs = +0.03; *p* = 0.72), MES (Rs = −0.03; *p* = 0.73), or MQS (Rs = +0.03; *p* = 0.68). Thus, we conclude that a decrease in the meibomian gland density does not influence meibomian gland disease. Similarly, we observed no differences in MGL between those eyes with distorted glands and those with no distortion (median: 25.5% versus 28.2%, resp.; *p* = 0.48). This may implicate that distortion of the glands does not contribute to meibomian gland loss.

## 4. Discussion

Recently, several research groups have focused their interest on characterizing meibomian gland morphology and its association with ocular surface diseases such as meibomian gland dysfunction [1–3, 13]. Meibography enables the visualization of the meibomian gland structure by retroillumination using an infrared filter, and this technique has become an important tool for understanding the nature of MGL and tracking the course of the disease [14–16]. In this study, we observed that meibomian gland atrophy is clearly associated with age. Our observations are in concordance with previous studies documenting that the aging process is accompanied with functional and morphological meibomian gland alterations [14, 17–21]. Postmortem investigations of human eyelid tissue revealed that aging human meibomian glands show decreased meibocyte differentiation and cell cycling [21]. According to those findings and our observations, the aging process is strongly believed to be one of the most influential risk factors of meibomian gland atrophy.

In parallel, there are several findings suggesting a strong correlation between meibomian gland alterations and sex [14, 16, 19, 20, 22, 23]. However, the results of studies investigating those associations are controversial. According to Den et al., a higher incidence of meibomian gland atrophy among men older than 70 years was observed, whereas no significant changes were observed in subjects under 70 years of age regardless of sex. Arita et al. similarly noticed evident changes of gland morphology in an elderly male group compared to a female group of the same age [14, 19]. On the contrary, Pult et al. observed a significantly higher incidence of meibomian gland morphological changes in a female group [20]. Following this report, data from a study by Ban et al. showed the mean length of meibomian gland ducts in males was significantly longer than that in females [16]. We found no differences between MGL and sex in our study group. This is in concordance with previous reports clearly documenting no relationship between meibomian gland atrophy and sex [24]. Interestingly, we documented better meibum expressibility and quality in males than in females, indicating that sex influences meibomian gland function. Thus, we cannot exclude the possibility that sex differences in MGD prevalence depended upon the MGD grade.

Our study also provided evidence regarding the influence of several systemic conditions on meibomian gland loss. For the first time, we report that MGL was significantly more prominent in smokers compared to nonsmokers. It is widely demonstrated that chronic smoking has a negative effect on the ocular surface and can affect some tear characteristics [25]. Smoking can contribute to the deterioration of the lipid layer in precorneal tear film [26], and the tear lipid layer showed significant slowing in spread over the tear film with a concomitant significant increase in tear evaporation rate in smokers [27]. Despite the evidence supporting the association of cigarette smoking with dry eye disease, to date, there has been no confirmation of these associations with regard to MG loss in the general population. We suppose that chronic ocular irritation associated with smoking may be responsible for the keratinization of the conjunctival epithelium. Indeed, Avunduk et al. reported evidence that tobacco smoke altered the conjunctival structure in rats by causing squamous metaplasia in the conjunctiva surface epithelial layer [28]. Thus, we cannot exclude the possibility that exposure to cigarette smoke induces hyperkeratinization of orifices and excretory ducts, thus blocking the expressibility of meibum and eventually resulting in acinar atrophy.

Accordingly, we provided evidence that postmenopausal women treated with hormone replacement therapy had an increased risk of abnormal meiboscore. This observation is consistent with laboratory studies demonstrating that estrogen and progesterone regulate meibomian gland metabolism and control gene expression and lipid production in these glands [29]. In a large cohort study on 25,665 postmenopausal women, hormone replacement therapy was shown to increase the risk of dry eye syndrome [30]. Similarly, we observed that participants on antiallergy medications were more likely to have abnormal meiboscores. Several reports have documented that the systemic use of antihistamines has been associated with increased risk of dry eye [1, 31]; however, little is known on the influence of such drugs on meibomian glands. Thus the exact manner through which antiallergy drugs result in MGL remains a focus for ongoing research.

Experimental studies revealed that high glucose is toxic for human meibomian gland epithelial cells [32]. Accordingly, several studies documented that diabetes mellitus was associated with MGD [22, 33]. Surprisingly, we found no association of MGL with systemic diseases such as diabetes mellitus, after adjusting for age and sex. Since meibomian gland loss was not evaluated in other studies, we cannot exclude the possibility that discrepancies between study results were due to the differences in methodology and in the criteria used to define MGD.

To date, there are no established objective diagnostic criteria for MGD. Arita and associates have suggested that an ocular symptom score, lid margin abnormality score, and meibography score can differentiate patients with MGD from the normal population. They reported that the ocular symptom score had the best predictive value, followed by the lid margin abnormality score and meiboscore [34]. Consistent with this report, we observed that meiboscore correlates with severity of presented symptoms and that the OSDI appeared to be an independent predictor of an abnormal meiboscore. Accordingly, the OSDI positively correlated with meibomian gland quality and expressibility scores in our study. Thus, our observations support the notion that MGD is a symptomatic condition and that severe morphological and functional abnormalities of the meibomian gland are accompanied with significant ocular discomfort. Unfortunately, due to the commonality of dry eye symptoms including aqueous deficient dry eye (ADDE) and MGD, available questionnaires are unlikely to differentiate between etiologically distinct disease entities. To define more specific eye symptoms associated with MGD, we analyzed the particular symptoms reported by the patient and their relation to meibomian gland dropout. We observed that a history of chalazion or hordeolum, experience of puffy eyelids upon waking, and foreign body sensation appeared to be independent predictors of an abnormal meiboscore. Similarly, Arita and associates observed that the frequency of one (foreign body sensation) of the 14 symptoms questioned was significantly higher in the obstructive MGD group than the ADDE group [35]. Thus, our results may have implications for the future development of more refined questionnaires that might have diagnostic power to differentiate patients with MGD.

There have been several studies that evaluated the correlations among meiboscore, dry eye symptoms, TBUT, Schirmer test, and lid abnormality score [14] as well as between meibomian gland loss and the lipid-layer pattern [20, 36]. However, the studies that estimated the correlations between meibum expressibility and quality and meibomian gland loss are rare. Arita and associates reported that the meibum score had a low power to differentiate patients with obstructive MGD from the normal population [34]. More recently, they documented that the meibum score differed significantly between patients with obstructive MGD and those with ADDE and recommended the meibum score as a relevant diagnostic parameter to enhance the reliability for differentiating between MGD and ADDE [35]. In the present study a positive correlation was observed between MG dropout and abnormal meibum quality and expressibility. These data support the concept that more available diagnostic procedures such as meibum analysis may be useful for verifying meibomian gland disease. Interestingly, we did not observe a correlation between the lid margin abnormality score and meibomian gland loss. There is considerable evidence that obstructive meibomian gland dysfunction may be recognized without obvious signs of ocular inflammation. With progression, MGD is likely to become symptomatic, and additional lid margin signs (e.g., telangiectasia) may be detected with the slit lamp [12]. The prevalence of the so-called nonobvious meibomian gland dysfunction appears to be high but significantly underreported. The clinical diagnosis of this condition is dependent on diagnostic meibum expression [37]. Thus, we conclude that expression of the gland and meibum assessment along with meibography are vital for an MGD diagnosis, specifically in patients where inflammation and other signs of the pathology are absent.

Interestingly, we found no association between MGL and MG distortion in our study. The exact mechanism underlying the development of MG distortion is unclear. Since increased meibomian gland duct distortion was observed in patients with perennial allergic conjunctivitis and contact lens-related allergic conjunctivitis, it has been speculated that inflammatory changes due to allergic reaction in the conjunctival tissue seemed to be the causative factor [38, 39].

Remarkably, the extent of MG dropout did not correlate with the tear film TBUT and the Schirmer test values in our study. These findings are in accordance with previous reports [2, 14, 19]. Accordingly, Arita and associates provided evidence that TBUT had relatively low power to differentiate MGD from normal subjects [34]. This may indicate that further research is necessary to understand the basis for symptoms in MGD and their relationship with dry eye syndrome.

Taken together, we conclude that aging process undoubtedly represents one of the major causes of meibomian gland dropout. Our data also show that postmenopausal hormone therapy, antiallergy drugs, and smoking are significant contributors to meibomian gland morphology. The results presented here indicate that an OSDI structured questionnaire as well as more defined investigation, including a history of chalazion or hordeolum, experience of puffy eyelids upon waking, and foreign body sensation, has diagnostic power to identify patients with meibomian gland loss. Accordingly, our results support the notion that other diagnostic procedures such as analysis of meibum quality and expressibility may be useful for verifying morphological changes of meibomian glands. Our findings extend current knowledge on the pathophysiology of MGD and may have implications for the future development of effective preventive measures against this disease.

## Figures and Tables

**Table 1:** Associations of ocular symptoms with meibomian gland loss (MGL) in 182 eyes of healthy volunteers.

Parameters	MGL (%) Mean ± SD		Abnormal meiboscore (stage ≥ 2)	
	Yes	No	OR (95% CI)^#^	*p* ^#^
Dryness	31.2 ± 11.6	28.9 ± 12.7	1.28 (0.66–2.496)	0.46
Foreign body sensation	32.7 ± 13.1	27.8 ± 11.0	2.5 (1.3–4.82)	**0.006**
Pain	32.0 ± 12.0	28.1 ± 12.1	1.79 (0.92–3.5)	0.09
Ocular fatigue	30.9 ± 11.4	27.9 ± 13.7	1.72 (0.84–3.55)	0.13
Blurred vision	31.3 ± 11.9	28.6 ± 12.4	1.43 (0.75–2.72)	0.27
Discharge	30.0 ± 10.3	29.9 ± 12.4	0.71 (0.23–2.22)	0.56
Epiphora	31.3 ± 12.4	27.6 ± 11.5	1.33 (0.66–2.62)	0.41
Symptoms' presence				
(i) On waking	28.8 ± 12.5	30.2 ± 12.2	0.47 (0.19–1.14)	0.09
(ii) At evening	31.9 ± 13.0	28.8 ± 11.7	1.65 (0.84–3.21)	0.14
(iii) During all day	28.4 ± 11.5	31.0 ± 12.6	0.84 (0.43–1.64)	0.61
Puffy eyelids on waking	33.3 ± 12.8	28.6 ± 11.8	2.42 (1.17–5.02)	**0.02**
Sticky sensation	29.2 ± 10.9	30.0 ± 12.4	0.62 (0.22–1.72)	0.35
History of chalazion or hordeolum	38.1 ± 10.8	29.0 ± 12.0	6.33 (2.08–19.26)	**0.001**

^#^Multivariate logistic regression model adjusted for age and sex with the specified parameter as the independent variable and abnormal meiboscore as dependent variable.

**Table 2:** Associations of systemic factors with meibomian gland loss (MGL) in 182 eyes of healthy volunteers.

Parameters	MGL (%) Mean ± SD		Abnormal meiboscore (stage ≥ 2)	
	Yes	No	OR (95% CI)^#^	*p* ^#^
Diabetes mellitus	41.9 ± 15.8	28.9 ± 11.4	2,82 (0,83–9,63)	0,096
Heart disease	34.5 ± 16.4	29.4 ± 11.5	1,15 (0,41–3,2)	0,79
Thyroid disease	35.1 ± 10.9	29.3 ± 12.2	2.04 (0.76–5.51)	0.16
Medications:				
(i) Antihypertensive drugs	32.6 ± 14.4	29.4 ± 11.7	1.44 (0.59–3.52)	0.42
(ii) Hormone replacement therapy^+^	38.3 ± 10.6	28.4 ± 11.5	5.72 (1.8–18.13)	**0.003**
(iii) Anticontraceptive drugs^+^			2,46 (0,62–9,76)	0,197
(iv) Antiandrogens^∧^	43.9 ± 21.8	29.2 ± 11.8	2.74 (0.2–36.82)	0.44
(v) Antidepressants	26.7 ± 10.1	30.1 ± 12.3	1,16 (0,24–5,49)	0,85
(vi) Antiallergic drugs	39.4 ± 12.9	28.4 ± 11.4	6.19 (2.39–16.05)	**0.0002**
Smoking	34.3 ± 15.4	28.4 ± 10.5	2.05 (1.01–4.14)	**0.04**
Computer use	28.3 ± 12.1	33.0 ± 12.0	0.8 (0.35–1.81)	0.59
Work environment:				
(i) Outdoors	27.8 ± 10.7	30.2 ± 12.4	0,58 (0,19–1,73)	0,32
(ii) Indoors with air conditioning	29.6 ± 11.1	30.1 ± 12.6	1,59 (0,78–3,24)	0,2
(iii) Indoors without air conditioning	29.9 ± 12.2	30.1 ± 12.4	0,97 (0,46–2,05)	0,94

^#^Multivariate logistic regression model adjusted for age and sex with the specified parameter as the independent variable and abnormal meiboscore as dependent variable.

^+^In the subgroup of women.

^∧^In the subgroup of men.
